# Neonatal Temporomandibular Joint Dislocation: A Case Report and Literature Review

**DOI:** 10.7759/cureus.40051

**Published:** 2023-06-06

**Authors:** Tyler A Le, Alison C Ma, Sean Clausen, Michele M Carr

**Affiliations:** 1 Medical Education, American University of the Caribbean, Cupecoy, SXM; 2 Otolaryngology, Jacobs School of Medicine and Biomedical Sciences, University at Buffalo, Buffalo, USA

**Keywords:** tmj anatomy, neonatal tmj, tmj dislocation, pediatric mandible, tmj morphology, temporomandibular joint (tmj) disorders

## Abstract

Neonatal temporomandibular joint (TMJ) dislocation is rare. The purpose of this study is to describe a case of neonatal TMJ dysfunction and to review the literature on this topic.

A six-day-old female was seen with both parents for evaluation of a dislocating jaw. Her mother had been breastfeeding successfully but noticed that there was a noticeable click every time the baby swallowed. Her jaw came out and down as she fed and then returned to the normal position. Over the last few days, her mother felt that only one side was involved as her jaw movement seemed asymmetrical. Her primary care physician had witnessed the click during the sucking reflex. The patient had a normal appearance and was otherwise healthy. The pediatric otolaryngologist observed deviation of the jaw toward the left with a palpable click upon mouth opening and spontaneous reduction with mouth closing. The symptoms resolved over the following month.

The literature review showed few cases of TMJ dislocation in infants, most of which described fixed dislocation related to vomiting or crying. Due to the development of the TMJ in infancy characterized by joint laxity and a flat mandibular fossa, malfunctioning of the hinge joint mechanism could be expected to be more common early in life.

## Introduction

The temporomandibular joint (TMJ) is a synovial, condylar, and hinge joint. The joint allows for sliding and hinge-like movements that ultimately allow for the mandible to move while articulating with the skull [1]. The function of the TMJ is complex with great importance for mastication as well as articulation. 

The TMJ is not completely mature at birth [1]. It is one of the last joints to develop in utero and can take years to fully develop postnatally. The joint at birth is more malleable due to the fossa, in which the head of the condylar process sits, being flatter and less developed in comparison to adults. This allows for hypermobility of the condylar process which can lead to dislocation [2].

TMJ dysfunction is a condition in which the joint is not articulating correctly. Dislocation occurs when the condylar head slips out of its position in the glenoid fossa and sits anteriorly [2]. Persistent or chronic dislocations can cause changes in the joint structure, which makes manual reduction more unlikely to be successful [3]. Persistent dislocations can cause the muscle to contract, which can lead to irreversible muscle fibrosis [3,4].

## Case presentation

A six-day-old female was seen with both parents for evaluation of dislocating jaw. Her mother had been breastfeeding successfully but noticed that the baby made a clicking noise with each swallow. Her jaw protruded anteriorly and inferiorly as she fed and then returned to the normal position. The mother continued in her report that over the last few days, her mother felt that only one side was involved as her jaw movement seemed asymmetrical involving only one side. The primary care physician had witnessed the click with the sucking reflex.

She, the patient, had been delivered by cesarean section at 38 weeks. Her birth weight was 6 lbs 5 oz and she was gaining weight appropriately. She required light therapy for jaundice, which was successful. Her history was otherwise unremarkable.

On examination, the head and neck appeared to be normal. The face was symmetrical. The jaw had a notable deviation to the left and made a palpable click when her mouth was opened. When she closed her mouth, the jaw was reduced to its normal position. The patient was monitored in the outpatient setting and the dislocation resolved after one month.

In the case of our patient, it is possible that trauma occurred during feeding, excessive crying, or delivery but no known trauma was recalled. Her TMJ dislocation spontaneously improved with time; therefore, a congenital etiology could not be ruled out given her young age.

## Discussion

TMJ dislocation in infants is very rare. TMJ dysfunction may be congenital or acquired, which can lead to dislocation. In the pediatric population, trauma is more common compared to non-traumatic causes [5]. Acquired dysfunctions could result from minor trauma such as a fall. Other causes such as excessive vomiting, crying, falls that impact the chin, or forceful feeding are rare [5]. At birth, the joint is flatter and may be easily susceptible to minor trauma. Here, we present a unique case of a newborn with a unilateral dislocation. To our knowledge, there are not many cases in the literature presenting TMJ dislocations in this age group.

**Figure 1:**
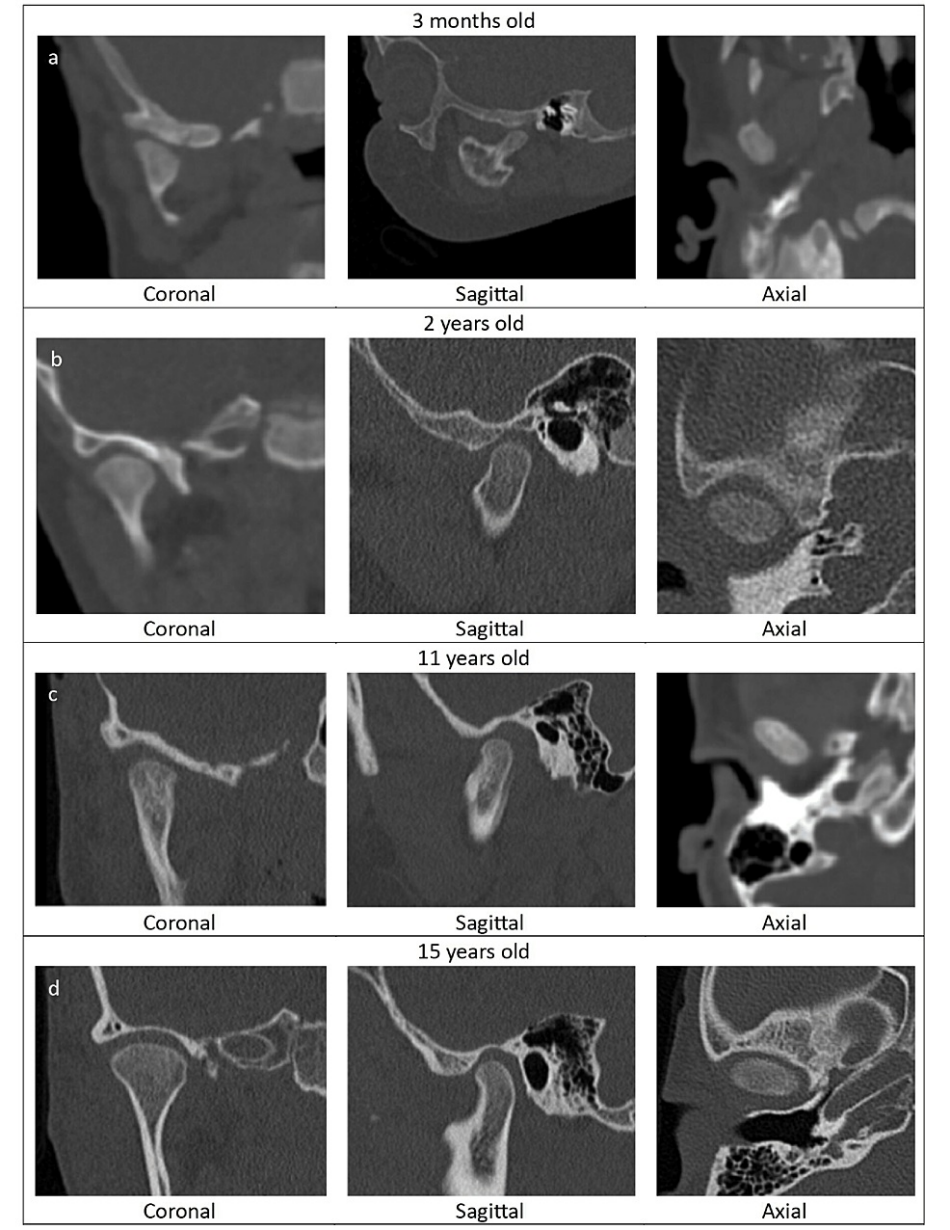
Computed tomography of the temporomandibular joint through childhood (a) CT head without contrast showing TMJ in the coronal (left), sagittal (middle), and axial planes (right) at three months old. The glenoid fossa is flat. (b) CT head without contrast showing TMJ in the coronal (left), sagittal (middle), and axial planes (right) at two years old. (c) CT head without contrast showing TMJ in the coronal (left), sagittal (middle), and axial planes (right) at 11 years old. (d) CT head without contrast showing TMJ in the coronal (left), sagittal (middle), and axial planes (right) at 15 years old. The glenoid fossa is concave making the joint more stable. CT: computed tomography; TMJ: temporomandibular joint

The TMJ is the last joint to develop in utero. At the eighth week of gestation, an area of mesenchymal blastema appears near the mandibular condyle and the glenoid fossa. At the 10th week, bone and cartilage begin to appear at the mandibular condyle [5]. The condylar and temporal blastema then forms around week 12. By week 14, a thin intermediate zone forms. At birth, the mandible is underdeveloped and small, and the TMJ is more malleable. Extensive remodeling takes place throughout the development of a child's growth through adolescence, and depending on the sex of the person, through early adulthood. The first mandibular growth spurt occurs at five to six years of age, beginning with expansion of the mandibular length, followed by the ramus height and corpus length. Mandibular growth stops two to three years following menarche in females, which is typically about 15 years of age. The maximum width of the mandible is reached in the late teens for females or early twenties for males. Width finalizes in early adolescence [2]. TMJ anatomy changes with growth are shown in Figure 1.

The literature review revealed only a handful of reports of TMJ dislocation in infants, most describing a fixed dislocation related to vomiting or crying [6-8]. Turgut et al. presented a case of a six-month-old child with TMJ dislocation with no known trauma [5]. The joint was eventually reduced under inhalation anesthetics. Cil et al. presented a unique case of dislocation discovered in-utero at 27 weeks gestation [9]. The patient also had a family history of TMJ dislocations, making congenital etiology highly likely. Whiteman presented a case of bilateral dislocation in a 10-month-old child due to vomiting [6]. It is logical to deduce that given the forceful nature of vomiting can serve as trauma to the TMJ. Painatt et al. reported a case of TMJ dislocation in an 18-month-old patient due to forced feeding [10].

TMJ dislocations are physiologically more possible in infancy and childhood because the joint is not as developed as it would be in other patient populations [1]. The glenoid fossa is flat, allowing for more mobility of the articular eminence. However, the articular eminence is not usually prominent enough for the dislocation to be maintained, resulting in spontaneous relocation, as in our case [9]. This is not to say that as the child progresses, the articular eminence will increase in inclination. The only factor known to increase the inclination is gender [11]. In cases where the dislocation is maintained, manual reduction under sedation can be performed. The other methods identified in manually reducing a TMJ dislocation are the Hippocrates method, the wrist pivot method, and the extraoral method [3]. All of these maneuvers articulate the mandible in a way to replace the articular eminence black into the glenoid fossa. More research would need to be done to determine the outcomes of using one method over the other.

Improper management of TMJ dislocation may lead to mastication, airway, speech, or cosmetic abnormalities. Improper management also increases the risk of chronic TMJ dislocations. Improper management or diagnosis can cause the dislocation to become persistent. Persistent dislocation, one that is maintained for 14 days or longer, can incur changes to the muscle leading to contracture and fibrosis. In this event, the only treatment option would be surgery [3,4].

## Conclusions

The TMJ is multifunctional and continues to develop postnatally. Neonatal TMJ dislocation is an uncommon condition that may improve with time as the joint structure matures and the glenoid fossa becomes less flat. It is not common for dislocations to occur in the pediatric population, but when it does occur, it is often attributed to trauma. Interestingly, even though trauma is cited as more common, the literature review demonstrated many case reports of non-traumatic causes as the reasons behind some of the pediatric TMJ dislocation. Treatment is recommended to prevent further sequelae. From the literature review, manual reduction is the first-line treatment while under sedation. But surgery remains an option for chronic or persistent dislocations.

## References

[1] Bender ME, Lipin RB, Goudy SL (2018). Development of the pediatric temporomandibular joint. Oral Maxillofac Surg Clin North Am.

[2] Kaplan AS, Assael LA (1991). Temporomandibular Disorders: Diagnosis and Treatment. https://books.google.co.in/books/about/Temporomandibular_disorders.html?id=VvNpAAAAMAAJ&redir_esc=y.

[3] Prechel U, Ottl P, Ahlers OM, Neff A (2018). The treatment of temporomandibular joint dislocation. Dtsch Arztebl Int.

[4] Pillai S, Konia MR (2013). Unrecognized bilateral temporomandibular joint dislocation after general anesthesia with a delay in diagnosis and management: a case report. J Med Case Rep.

[5] Turgut NF, Özdemir D, Mehel DM, Akgül G, Özgür A (2023). Bilateral temporomandibular joint luxation in a 6-month-old child: case report. Cranio.

[6] Whiteman PJ, Pradel EC (2000). Bilateral temporomandibular joint dislocation in a 10-month-old infant after vomiting. Pediatr Emerg Care.

[7] Demirtas Y, Kucukoduk I, Oren AC, Celebi C (2005). Temporomandibular joint dislocation in an infant after vomiting. Plast Reconstr Surg.

[8] Cascarini L, Cameron MG (2009). Bilateral TMJ dislocation in a 23-month-old infant: a case report. Dent Update.

[9] Çil AS, Bozkurt M, Bozkurt DK (2014). Intrauterine temporomandibular joint dislocation: prenatal sonographic evaluation. Clin Med Res.

[10] Painatt JM, Veeraraghavan R, Puthalath U (2017). Temporomandibular joint dislocation in an 18-month-old child. Contemp Clin Dent.

[11] İlgüy D, İlgüy M, Fişekçioğlu E, Dölekoğlu S, Ersan N (2014). Articular eminence inclination, height, and condyle morphology on cone beam computed tomography. Sci World J.

